# Cognitive testing of a survey instrument to assess sexual practices, behaviours, and health outcomes: a multi-country study protocol

**DOI:** 10.1186/s12978-021-01301-w

**Published:** 2021-12-19

**Authors:** Lianne Gonsalves, Erin C. Hunter, Vanessa Brizuela, Joseph D. Tucker, Megan L. Srinivas, Evelyn Gitau, Catherine H. Mercer, Nathalie Bajos, Debbie Collins

**Affiliations:** 1 UNDP-UNFPA-UNICEF-WHO-World Bank Special Programme of Research, Development and Research Training in Human Reproduction (HRP), Department of Sexual and Reproductive Health and Research, World Health Organization, Avenue Appia 20, 1211 Geneva, Switzerland; 2 Sydney School of Public Health, Faculty of Medicine and Health, The University of Sydney, Sydney, Australia; 3 Faculty of Infectious and Tropical Diseases, London School of Hygiene and Tropical Medicine, London, UK; 4 Institute of Global Health and Infectious Diseases, University of North Carolina, Chapel Hill, USA; 5 African Population and Health Research Center, Nairobi, Kenya; 6 Centre for Population Research in Sexual Health and HIV, Institute for Global Health, University College London, London, UK; 7 Institut National de la Santé et de la Recherche Medicale (INSERM), IRIS-EHESS, Paris, France; 8 NatCen Social Research, London, UK

**Keywords:** Sexual health, Qualitative research, Reproductive health, Measurement, Cognitive interviewing

## Abstract

**Background:**

Population level data on sexual practices, behaviours and health-related outcomes can ensure that responsive, relevant health services are available for all people of all ages. However, while billions of dollars have been invested in attempting to improve sexual and reproductive health (including HIV) outcomes, far less is understood about associated sexual practices and behaviours. Therefore, the World Health Organization embarked on a global consultative process to develop a short survey instrument to assess sexual health practices, behaviours and health outcomes. In order for the resulting draft survey instrument to be published as a ‘global’ standard instrument, it is important to first determine that the proposed measures are globally comprehensible and applicable.

This paper describes a multi-country study protocol to assess the interpretability and comparability of the survey instrument in a number of diverse countries.

**Methods:**

This study will use cognitive interviewing, a qualitative data collection method that uses semi-structured interviews to explore how participants process and respond to survey instruments. We aim to include study sites in up to 20 countries. The study procedures consist of: (1) localizing the instrument using forward and back-translation; (2) using a series of cognitive interviews to understand how participants engage with each survey question; (3) revising the core instrument based on interview findings; and (4) conducting an optional second round of cognitive interviews. Data generated from interviews will be summarised into a predeveloped analysis matrix.

The entire process (a ‘wave’ of data collection) will be completed simultaneously by 5+ countries, with a total of three waves. This stepwise approach facilitates iterative improvements and sharing across countries.

**Discussion:**

An important output from this research will be a revised survey instrument, which when subsequently published, can contribute to improving the comparability across contexts of measures of sexual practices, behaviours and health-related outcomes. Site-specific results of the feasibility of conducting this research may help shift perceptions of who and what can be included in sexual health-related research.

**Supplementary Information:**

The online version contains supplementary material available at 10.1186/s12978-021-01301-w.

## Plain language summary

Sexual and reproductive health receives a lot of attention, globally. Much of this attention focuses on research and clinical services to address health outcomes. Behavioural issues known to be closely linked to health outcomes—that is people’s sexual activity and sexuality—tend to be overlooked. In many countries, there is very little data about people’s sexual practices and behaviours. If this information were available, then sexual and reproductive health programmes could be planned to better meet the needs of populations.

Therefore, the World Health Organization developed a survey instrument which can be used to ask people around the world about their sexual practice and behaviours. The protocol described here will test the draft survey instrument in different countries to make sure that people from diverse backgrounds understand each survey question in a comparable way.

The study uses ‘cognitive interviewing’, a qualitative method where researchers ask participants about their thought processes in reading a question and providing an answer while they complete a survey. Up to 20 countries will participate in this study, with five or more completing the research at a time in each study wave. Country teams in each wave will translate the survey instrument (if necessary), conduct the cognitive interviews, revise the instrument based on the findings, and if needed, conduct more interviews in their setting with the revised survey instrument.

The result of this research will be a survey instrument that has been tested in a variety of languages and settings and is ready to be used in different contexts, globally.

**Supplementary Information:**

The online version contains supplementary material available at 10.1186/s12978-021-01301-w.

## Background

Sexual health and wellbeing is an integral part of overall health and wellbeing [1]. Achieving the “right to the highest attainable standard of health” includes the ability to have safe and consensual sex. One central tenet of providing adequate, quality information and relevant services in any area of health is to have prior understanding of the existing related practices and behaviours of the population. Governments, private companies, and donors have invested billions of US dollars to strengthen sexual and reproductive health and rights (SRHR), including HIV, services [2, 3], and an SDG target commits to ensuring universal access to sexual and reproductive health services by 2030 (SDG3.7). Yet, due to their sensitive, sometimes stigmatizing nature, the practices and behaviours underpinning the need for these services are less well understood. Issues to do with sexuality and sexual activity are often overlooked, marginalized, or neglected, which adversely affects the availability and use of relevant data worldwide.

To date, there are a few rigorous surveys on sexual practices and behaviours that have been conducted in high-income countries at sub-national or national levels (e.g. Britain’s National Surveys of Sexual Attitudes and Lifestyles [4] or France’s Contexte de la Sexualité en France [5]). Additional surveys have asked about sexual practices and behaviours across multiple countries. However, these range in quality (for example in terms of their representativeness [6]), the detail that they capture on sexual practices and behaviours, and the demographics of their target population [7, 8]. Together, these data provide a patchy, but important base understanding of *some* sexual practices and behaviours for *some* populations at *specific* stages across the life course.

Robust population-level data on sexual heath are needed to ensure adequate services are available for all persons across the life course, recognizing the different SRHR issues that arise throughout life. Additionally, sexual health-related data have important implications for: identifying and challenging gender and social norms (e.g. understanding what constitute accepted sexual and intimate partner (mis)behaviours and expressions); decoupling specific sexual practices from certain populations (e.g. the perception that anal sex is a practice only among men who have sex with men (MSM) results in scientifically-discredited and human rights-violating forced anal examinations in certain countries where same-sex activity is criminalized [9]); and providing relevant sexuality education and information which is responsive to actual practices (e.g. provision of sex/sexuality-related information to older persons is often limited, with the false assumption that they have stopped engaging in sexual activity [10]).

In 2019, WHO’s Department of Sexual and Reproductive Health and Research, which includes the UNDP/UNFPA/UNICEF/WHO/World Bank Special Programme of Research, Development and Research Training in Human Reproduction (WHO/HRP) was funded by the Wellcome Trust to review available, quality nationally-representative sexual health/practices data from around the world (from sources described above) and determine what was needed to improve existing (or generate new) data. In June 2019, WHO/HRP convened a meeting of experts in sexual health research and data analysis, as well as experts with specific experience in conducting nationally-representative surveys on sexual health/practices. Participants reviewed the available data and confirmed that their heterogeneity made cross-national or global comparisons difficult, unless costly, complex and time-consuming indicator harmonizing procedures were implemented. Even within well-resourced national surveys, survey leads approached identical sexual health/practices thematic areas with different questions, response options, or time-points measured.

Meeting participants identified a need for a global standard instrument for assessing sexual health-related practices and behaviours in a consistent manner. Participants representing national surveys in France, Australia, Britain, Finland, and Slovenia, indicated that, were such an instrument available, they would seek to incorporate these measures into their own surveys to enable them to participate in cross-national comparisons. Additionally, participants indicated that other sexual health/practices researchers would benefit from a ‘go-to’ short survey instrument with standardized measures that could be incorporated into their study instruments. This would promote researchers measuring sexual health and practices consistently and in line with best practice.

Subsequently, a multi-stage, and globally consultative process to develop this short survey module was initiated and ran from September 2019 to January 2021. This process has been described in full elsewhere [11], and included a global call for measures, an in-person hackathon and modified Delphi exercise to develop the draft instrument, review by WHO and external experts, and a final public comment period. The development of this instrument, drew on the expertise of researchers from all six WHO regions, and used existing measures from validated survey instruments. Criteria for included measures is detailed in Box 1. A brief overview of the development process is also included as Additional file 1.

**Box 1:** Qualities of the short survey module of sexual-health related practices, behaviours and outcomes

This survey was meant to capture *priority* sexual health related measures and have the following qualities:
• ~ 10 min (average) completion time
• Appropriate for general population (age 15+)
• Consist of stand-alone measures (scales or indices are discouraged)
• Included questions have been implemented in existing surveys (measure creation is discouraged)

Ideally, a final instrument would be able to be easily localized with minimal content modifications between sites, so that data collected from different sites can be compared. However, many of these survey measures were established in high-income settings. This draft survey instrument cannot be published as a ‘global’ standard instrument for assessing sexual health-related practices and behaviours without first determining that its measures (1) are globally comprehensible and (2) assess the intended constructs consistently and correctly when translated and implemented in different settings.

As such, this protocol describes a proposed study to conduct cognitive testing of the draft instrument (Additional file 2) among members of the general population in diverse low- and middle-income as well as high-income settings.

### Problem statement, aims and objectives

The aim of this particular research study is to refine a standard instrument, in English and other language versions, by testing it in a variety of demographic cross-sections of the general population (e.g. older persons, persons in rural areas), worldwide. This will increase the global applicability and utility of this instrument. Specific outputs include:

- A standard set of sexual practice-related questions that have been tested in different languages across a variety of geographic and cultural environments.
- A process for adapting the survey instrument to a new setting (including translation), which can be replicated in the future by researchers wishing to incorporate the instrument into their sites.
- Recommended implementation ranges (for example, enabling WHO/HRP to provide an age range among which the tool can be implemented).

Cognitive interviewing will be used to determine whether target audiences (cross sections of the general population) are willing and able to answer the instrument’s questions. It will also determine whether questions are interpreted (understood) by the target population in the way intended.

This is a core protocol and lacks site-specific information. The ‘Discussion’ section briefly describes the separate but complementary process by which research sites have been identified.

## Methods

### Study design

**Fig. 1:**
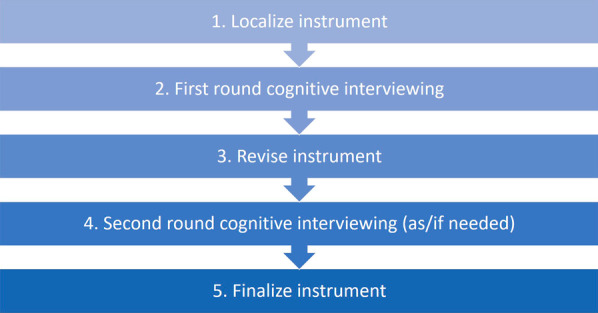
Process for testing survey instrument

This protocol is intended to determine the global acceptability and applicability of a survey instrument designed to assess sexual health-related practices, behaviours, and outcomes. The complete process consists of several steps, as outlined in Fig. 1.

**Fig. 2:**
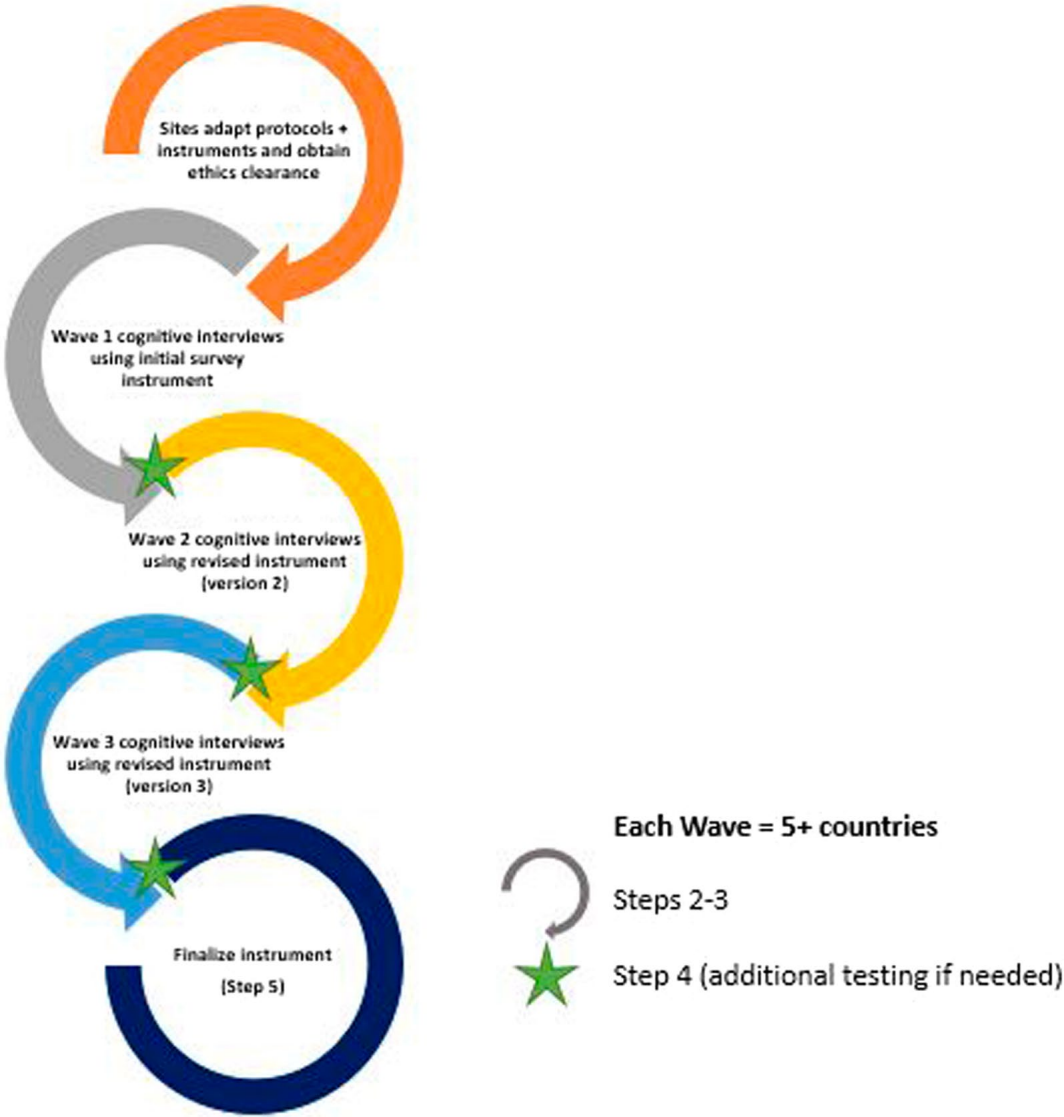
Wave approach to implementing protocol

The steps described in Fig. 1 will run simultaneously in the study sites within a single wave of 5+ countries. A total of three ‘waves’ are envisioned, with 20 study sites in total. After all sites have completed localized translations of the survey instrument and obtained local ethics approvals, the first wave will complete Steps 2–3. The revision (Step 3) will use data from all the sites in that wave. If necessary, one or multiple sites in that wave will begin Step 4, as the second wave begins Steps 2–3. The instrument will be finalized after three waves of countries have completed at least one round of cognitive interviewing (Steps 2–3). Figure 2 further describes this process.

Steps 1–5 are described briefly below (and in more detail in relevant sections, as indicated).

1. Localize instruments:
  Each participating site will translate the English-language core instrument into a local language [See ‘Data collection method/s’ section for details].
2. First round cognitive interviewing:
  Cognitive interviewing is a qualitative method which enables researchers to investigate participants’ thought processes as they encounter and develop a response to a survey question (see Fig. 3, developed from Tourangeau, 1984 [12]). Through this process, researchers can determine whether questions are being interpreted by participants as intended by survey authors [13]. Similarly, through cognitive interviewing, researchers can identify potential sources of response error when the survey is administered, including: complex design; or inappropriate/suboptimal wording, response options and/or order [14]. When survey instruments are meant to be available in multiple languages, cognitive interviewing also provides an opportunity to test whether problems arise due to translation error, factors influencing interpretation in different contexts, or from question design issues in the source instrument [15, 16].

**Fig. 3:**
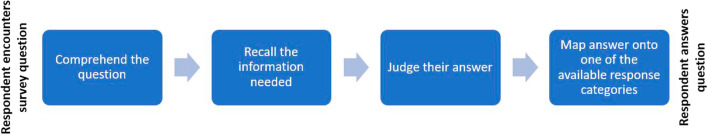
Cognitive process for responding to a survey question ( adapted from Tourangeau, 1984)
  Finally, for instruments that are meant to be implemented cross nationally, cognitive interviewing can help to establish the “cultural portability” of constructs across countries, and therefore the subsequent ability to compare data collected between countries [14]. As described by Willis, these so-called ‘cross-cultural cognitive interviews’ (CCCI)
  determine whether the different questionnaire versions illustrate the key property of *cross-cultural equivalence*; that is, whether the range of interpretations associated with the evaluated items varies acceptably between cultural or language groups, given the survey measurement objectives [17].
  Conventionally, cognitive interviews are conducted using either ‘think aloud’ or ‘probing’ techniques to elucidate participants’ thought processes as they complete a given survey instrument. When using the ‘think aloud’ technique, an interviewer asks participants to verbalize their thoughts as they are formulating a response, with the interviewer intervening very little otherwise. In ‘probing’, the interviewer has a more active role, asking targeted questions and follow-up probes to understand a participant’s interpretation of the question and reason for their selected response [13]. The ‘think aloud’ technique has proven challenging to implement across certain cultural contexts and so verbal probing is more suited to CCCIs [17], though participants spontaneously sharing their thought processes is never discouraged.
  The first round of cognitive interviews will involve 24–32 individuals in most country sites (with a maximum of 50 in any site) [See ‘Data collection method/s’ section for details].
3. Revise instrument:
  The data produced by cognitive interviews may identify sources of error and bias, as well as insight into participants’ lives (which may factor into the error or bias in their responses) [18]. This information feeds into the revision of the survey instrument [17]. If participants interpret the question as intended, it affirms the question’s construct validity [18].
  Revisions to the instrument will be based on the findings not only from a single study site but also through a comparison of findings across sites [See ‘Data analyses’ section for details].
4. [OPTIONAL] Second round cognitive interviewing:
  This second round will be completed with up to 10 individuals using the revised instrument.
  Step 4 remains optional, rather than required, as the iterative rounds of survey testing and refinement (described below and in Fig. 2), will ensure that revisions of the instrument continue to be tested in subsequent sites. A second round of cognitive interviews could be triggered in the case of any of the following:
  Step 2 reveals significant translation error that is, problems with *that site’s version of the survey instrument*.
  Step 2 reveals source survey instrument design issues, for example Likert-type response options are not understood in *that site* [19], so an alternate set of responses need to be developed and retested in those as well as future sites.
  Step 2 leaves certain question routes untested/undertested e.g. those around having multiple sexual partners.
5. Finalize instrument:
  Data will be analysed at country-level and then across countries within a given wave [See ‘Data analyses’ section for details].

### Study settings

It is important that cognitive testing for this survey instrument is conducted among the instrument’s intended target audience (the general population) across a variety of cultures. Geographically, 20 countries will participate: with 2–4 envisioned from WHO’s Americas Region; 4–8 total from WHO’s African Region and Eastern Mediterranean Region; 1–2 from WHO’s European Region; and 4–6 total from WHO’s Western Pacific Region and South-East Asia Region. Countries will be predominantly, but not exclusively, low- and middle-income countries (LMICs) due to the lack of existing sexual practices related data in LMICs as compared with high-income countries. Importantly, the collective set of countries will reflect a range of political and cultural openness regarding sexual activity and sexuality.

The success of cross-cultural cognitive interviews is facilitated when research processes take place simultaneously so that findings can be compared across sites. This provides insight as to whether a given issue is unique to a certain site or shared by people of similar demographics in different countries. Therefore, respecting the different speeds of research processes in different countries, including protocol development and approval, the 20 countries will be bundled into waves of five or more countries, as depicted in Fig. 2.

This core, generic protocol will be adapted in each country for the specific sites. In addition to presenting rural/urban, socio-economic, and educational (e.g. average years of schooling) demographics of the country, site-specific protocols will describe the presence of sexual health-related laws and policies. This would include, for example, laws which: criminalize same sex activity; enable/restrict youths’ ability to engage in sexual activity or access SRHR services; and criminalize intimate partner violence (including within marriage). Additionally, site-specific protocols will present sex-disaggregated statistics (where available) on average age at first sex, average age at first union, as well as contraception prevalence. This will provide additional, SRHR outcome-related insight.

It should be noted that many of the above statistics only exist at the national level. Cognitive interviewing, by comparison, will involve relatively few individuals recruited from a specific part of the country. As such, subnational indicators will be used where available.

### Study participants and sampling

This study’s target population will be the general population, defined as those aged 15 years and over. Sexuality is an element of the human experience which exists from birth. However, given that this instrument focuses heavily on present/previous sexual activity, the proposed lower age range of fifteen should capture a majority (though not all) sexually active persons. In some cases, it may be necessary for a site to restrict recruitment of participants to a narrower range of ages (e.g., 16+ or 18+, etc.) to ensure adequate protection of participants under local laws and/or customs. There is no upper age limit proposed for this protocol, as sexual activity continues throughout the life course.

Between the first and optional second round of cognitive interviewing, all site-specific protocols will conduct 34–42 interviews with the general population. Sites will conduct 24–32 interviews in the first round of cognitive testing. Within the target population it is important to ensure heterogeneity in terms of characteristics that may affect the way in which the questions may be understood, such as age or sex. Therefore, each site will aim to obtain approximately equal numbers of male and female participants across four general population age groups: 15–19, 20–24, 25–59, and 60+.

A minimum of 10 of these 24–32 participants should ideally be recruited from rural communities. Additionally, each site can choose to recruit 4–8 participants from specific population groups that are either (1) more difficult to reach or where additional, special outreach may be required; or (2) too small to reliably reach through general recruitment measures. Examples of these specific population groups may include:

- Persons living with disabilities.
- Lesbian, gay, bisexual, and/or transgender individuals.
- Persons who have had more than one sexual partner in the last year.

Rural and ‘subset population group’ participants can be distributed across the proposed age/sex matrix, shown in Table 1.

**Table 1:** Age/sex matrix to obtain 24–32 ‘general population’ interviews

	15–19	20–24	25–59	60 +
Males	3–4	3–4	3–4	3–4
Females	3–4	3–4	3–4	3–4

If sites have particularly strong existing research ties with any of the groups above, they may choose to collect up to a maximum of 20 additional interviews from persons who are part of this population.

As is described in the ‘Data collection method/s’ section below, research sites will have an option to make sensitive modules within the instrument self-administered, along with the accompanying probes. Self-administered modules reflect current practice in similar surveys [20]. Self-administered sections are perceived to minimize discomfort and social desirability bias on the part of respondents who may not feel comfortable verbally engaging with an interviewer on sensitive, sex-related questions.

Self-administered components also mean that participants need to be able to read a question to themselves and write/enter a response. Therefore, in settings where survey instruments and/or probes are implemented as self-complete, site-specific protocols will introduce a literacy requirement as part of their eligibility criteria.

Sampling in all sites will be nonprobability based and can include snowball sampling or purposive sampling. Purposive sampling, in line with what has been observed in several CCCI studies [17, 21], will rely on one or more site-specific recruitment channels, for example: newspaper or online advertisements, including on social media platforms; flyers; or in-person outreach at markets, community and/or health centres, etc. In sites where snowball sampling is used, recruiters will provide initial purposively selected study participants with study information and research team contact information to share with other potential participants in their network. Those interested will be able to follow up independently with the study team should they wish to participate. Each site will determine the method most appropriate for reaching their target populations.

In all sites, recruitment materials will include contact information for the study team. When a potential participant contacts the study team, whether in-person or by phone, messaging platform, online form, or email, a study team member will screen the participant for eligibility and set a time and place for an interview.

Each site will determine whether interviews will take place in-person or virtually via videoconferencing software. These decisions will be based on the current status of COVID-19 pandemic restrictions and the general population’s access to electronic and mobile devices. ‘Virtual’ interviews will require video and voice (rather than voice alone), so that interviewers are able to show cue cards, and better gauge body language and responses. A participant can request to stop the use of video at any point during an interview while continuing with sound only. The interviewer will keep a record of this in their notes.

Sites will also have the option to add self-administered web-based surveys; essentially, an online survey with no interaction with the research team. In some settings, web probing has been found to generate comparable findings to cognitive interviews [22]. However, the success of web probing is context specific (that is, in sites where such survey research is common, and access to and comfort with web-based technology is widespread). Therefore, web-based surveys will supplement rather than replace virtual/in-person interviews. These interviews are not included in the study participant numbers—they will be included in site-specific protocols, should a site decide to include them. Additional file 3 provides a COVID-19-inspired overview of approaches to in-person, virtual, and web-based cognitive interviewing and the relative strengths and weaknesses of adopting one of these approaches in a study taking place during a pandemic.

Prior to the start of the interview, the researcher will provide the participant with a study information sheet in the local language and walk the participant through each part of the sheet before obtaining written or oral/verbal consent. Whether consent is written or oral/verbal will be determined based on literacy, local conventions for research on sensitive topics, and/or whether the data collection will take place face-to-face or remotely. Table 2 provides details on the circumstances under which a site may opt to obtain written versus oral/verbal consent.

**Table 2:** Circumstances for obtaining oral/verbal consent versus written consent

	Examples reasons for use	Example process for in-person data collection	Example process for remote data collection
Oral/verbal consent	May be used in the case(s) of low literacy; where there are site-specific cultural/political concerns with signing contract-like documents; where obtaining oral consent is the convention (with local IRB approval) for researching sensitive topics; and/or instances where data collection is conducted remotely and written consent is not feasible or is overly burdensome on participants	Participant information sheet will be provided to participant and explained by researcher. Researcher will either audio record the consent process (with permission) and/or sign a record of oral consent form after answering any questions the participant had, confirming that the participant freely gave oral consent	Participant information sheet will be provided to participant either by mail in advance of the interview or electronically (e.g. email, messenger service, or web form). At the time of the interview, the researcher will verbally explain the participant information sheet. Researcher will either audio record the consent process (with permission) and/or sign a record of oral consent form after answering any questions the participant had, confirming that the participant freely gave oral consent
Written consent	Written consent will be sought in cases other than those cited above	Participation information sheet with consent form will be provided to participant and explained by researcher. Participant will sign form to indicate consent and researcher will sign confirming that the participant was given an opportunity to ask questions, all questions were answered, and the participant freely gave consent	Participation information sheet with consent form will be provided to participant either by mail in advance of the interview or electronically (e.g. email, messenger service, or web form). At the time of the interview, the researcher will verbally explain the participant information sheet and consent form. Participant will sign form to indicate consent. Depending on context and resources available to the participant, this may be done with a digital signature and sent electronically back to the researcher; or a digital checkbox indicating consent; or the participant may print, sign, and scan the form and return to the researcher. Researcher will sign confirming that the participant was given an opportunity to ask questions, all questions were answered, and the participant freely gave consent

For individuals under the age of majority, where appropriate and in line with local institutional review board requirements and laws, the participant’s written consent will be obtained with parental/guardian consent waived [23].^1^ In sites where waiving the requirement of parental/guardian consent is not allowed according to local institutional policy or laws, site-specific protocols will describe the process of obtaining parental/guardian consent and participants’ assent if they include participants under the age of majority.

All participants will be provided with a copy of the consent form as well a separate page containing a short description of the study, study team contact information, and links to relevant, local SRHR-related online resources and/or services, to take with them.

Following revision of the instrument (Step 3) based on the findings of all sites in that wave, a study site may choose to conduct up to an additional 10 interviews (Step 4). Eligibility criteria and recruitment procedures will remain as described above.

### Sample size calculation

The sample size described above is an estimation of the number of interviews required to capture a wide range of reactions to the instrument. A general practice is for cognitive interviews to be conducted in iterative rounds of 5–15 [13]. Factoring in the complexities of comparing findings across countries, the sample size increases. As such, each site protocol will have a total maximum sample size of between 24 and 60 (24–50 from the first round of interviews, as well as 10 additional interviews for an optional second round). With 20 study sites envisioned, the global sample size will be between 480 and 1240, which is in line with similar CCCI studies [17].

### Data collection method/s

#### Step 1: localize instruments

Each site will first translate the English-language draft survey instrument and semi-structured cognitive interview guide into the local language, and then back-translate it into English. The individual(s) translating the core instrument will be different from the individual(s) back-translating to English. The two versions will then be compared for discrepancies and discrepancies will be discussed by the two translators with a third member of the team adjudicating.

#### Step 2: first round cognitive testing

Trained researchers will conduct cognitive interviews by administering the draft survey instrument to an individual and collecting verbal and nonverbal information about *how* the individual interprets the question and arrives at a response [24]. Researchers will use a semi-structured interview guide with suggested probes for each question being tested. Probing can take place after each question is answered or after all questions in a given section have been answered [18]. Probes are open-ended, with scope for interviewers to use unscripted elaborative and expansive probes to further explore participants’ understanding of the questions and reactions to them [18]. Probes may be answered verbally or in writing, based on the participant’s comfort. Additionally, the interviewer can probe on specific questions if they note the participant looking confused, contemplative, uncomfortable or otherwise having a noticeable ‘reaction’ (verbal or physical) to a question.

This survey instrument is envisioned to ultimately be delivered using a combination of interview-administered and self-administered modules. However, the goal of the present study is to create an interview environment where every participant (1) understands what is being asked of them; and (2) is comfortable enough responding to be able to provide the research team with usable data as described above. As such, probes may be answered verbally, or in writing (with paper provided by the interviewer or on a digital device), based on the participant’s comfort. Each site will have the opportunity to implement the interview in one or more of the following formats:

1. Parts of the survey (Additional file 2) that are self-administered are completed by the participant themselves, along with relevant interview probes. This would involve the participant entering survey and probe responses by hand (either handwritten on a paper instrument, or typing a response on a digital device).
2. Parts of the survey that are self-administered are completed by the participant themselves by hand, but ALL of the interview probes are administered by the interviewer.
3. ALL of the survey and ALL of the interview probes are administered by the interviewer.

Sites will specify the option(s) they intend to make available in their setting.

Probes will explore:

- Comprehension of key terms.
- Whether participants are able to recall the information requested and whether they constrained their thinking to the time period described.
- Whether answer options are complete and used appropriately.
- Whether participants feel that they (and others) could give an honest answer.
- Whether participants perceived the questions to be phrased in a sensitive manner.

All cognitive interviews will be conducted in private and audio-recorded, with the participant’s consent. Interviews will be conducted in the presence of only the data collector, where possible. In previous cognitive interview studies, participants have been given a gift voucher or other modest ‘token of appreciation’ as a thank you for their time [25]. Each site will offer something similar, not intended to exceed the relative equivalent of USD 20, or an amount deemed appropriate for this type of research for local research teams and ethics review boards/committees.

#### [OPTIONAL] Step 4: second round cognitive testing

In select sites, a revised instrument may undergo cognitive testing in a second round of interviews. The data collection procedure will be the same as described above.

### Data analyses

Within-site data analysis will focus on summarizing the findings from each interview, adopting a pragmatic approach similar to that implemented as part of cognitive testing for Natsal-4 [20]. All countries will be provided with an analysis matrix, which captures responses to each test question and corresponding probes for each individual. In this way, data from a given site can be read horizontally as a complete summary of one participant’s interview, or vertically capturing all participants’ responses to the same question/probes.

Each country will send its matrix to WHO/HRP, who will lead the cross-country review of the findings, according to the CNEST (Cross National Error Survey Typology), developed as part of a similar multinational survey instrument design process [16]. CNEST categorizes error according to three classifications: (1) poor source question design; (2) translation problems resulting from either (a) translator error or (b) source question design (vague quantifiers); and (3) cultural portability. WHO/HRP will review findings across all countries in a ‘wave’ together and make preliminary identifications of questions that need to change in the source survey instrument. These findings will be discussed in a half day joint analysis meeting (JAM) with the PIs and/or study coordinators of that wave. The JAM will cover:

- Findings which remain unclear/in conflict within or across sites.
- Proposed modifications to the survey instrument.
- The need for one or more sites in the wave to test the revised survey instrument.

### Data management and data access

All study results will be kept confidential by the team in either password-protected files for electronic data, including audio files, or locked cabinets for interview notes on paper. Only approved team members will have access to study results.

Labelling data: A master list will be maintained that includes ID numbers that are uniquely assigned to each participant. Interview notes, audio files, consent forms, and other interview data will be labelled only with these ID numbers.

Storing paper documents: Master ID lists and informed consent forms (which contain the participant’s identifying information) will be stored in a locked cabinet that is separate from any other study material. Any other hard copy documents that contain study results will be stored in a locked cabinet that is accessible only to key study personnel.

Storing digital data: Digital files (audio, data analysis, interview notes, completed survey instrument) will be stored securely on a password-protected computer and on password-protected cloud storage such as Dropbox. Access to files on cloud storage will only be granted to select research staff who will be participating in the data analysis. The original interview audio recordings will be destroyed after 2 years, while additional study materials will be destroyed after 1 additional year.

Each site will be responsible for maintaining the content of each interview: the audio file of interview and any written notes, and a record of the completed survey instrument. Within-site analyses, as described above, will result in a completed matrix file which is shared to WHO/HRP. WHO/HRP will pool these files, as described above, generating one master matrix that contains data from all sites in a given wave. Site PIs will have access to this file for the purposes of the joint analysis meeting.

### Ethical considerations

Some specific ethical challenges that this protocol presents are described below, along with details as to how the research partners will address these.

First, the research subject matter (sexual practices and behaviours) is sensitive and may be considered a taboo research subject that could cause participants discomfort. This could be a concern for ethics review committees. In response, all site-specific protocols will make a clear case as to *why* this kind of research is important locally, and clearly indicate how participant comfort will be maintained (e.g. in addition to obtaining informed consent, repeating to participants that they may stop the interview at any time, are under no obligation to respond if uncomfortable, etc.).

Second, cognitive testing may reveal certain ongoing/past traumatic sex-related experiences on the part of participants. In response, all site-specific protocols will provide participants with information about how to access local counselling and/or support services. Participant information sheets will specify that interviewers can suggest referrals to participants when they feel like they or someone around them may be at risk. In the event that reliable services are not available, interviews will not be conducted in that area.

Third, mandatory reporting laws may place researchers in a compromising position where legal reporting obligations conflict with their ethical obligations to put the welfare of the participant first. This could include reporting of activity criminalized in the country, including commercial sex work, or same-sex activity. It could also, however, include age-specific legislation which may require an adolescent participant desiring SRHR services to have the consent of a parent or guardian (a breach of the participant’s confidentiality).

In response, as part of protocol development, each site will identify any mandatory reporting laws. Each site will determine if exemptions from reporting for the purposes of research already exist or can be obtained [26]. If this is possible, site-specific protocols will specify this. In the event that this is NOT the case, research sites will consult with local ethics review committees and civil society and/or advocacy groups for advice on how to balance these obligations, while keeping the welfare and confidentiality of the participant a primary objective [23, 27]. Site-specific protocols will indicate the results of these discussions. In the event that a satisfactory, participant-centred solution is not possible, relevant questions will be dropped from testing in that site and/or may choose to restrict recruitment criteria to ensure better protection of participants (e.g. a site where sex outside marriage is illegal and the minimum age of marriage is 16 may choose to only recruit participants 16+ who have been married).

Finally, in some cases, even where mandatory reporting requirements do not exist/are waived, cognitive testing of some questions may put certain participants (particularly members of already-marginalised LGBTI groups) at risk in settings where specific sexual behaviours are criminalised. In response, as described above, each site will determine, in consultation with local groups and based on review of local laws and policies and social norms, whether asking any questions could have serious adverse consequences for participants. The presence of any relevant laws/policies will be clearly noted in the ‘Study setting’ section of the site-specific protocol, and in the event that concerns (described above) cannot be mitigated, any relevant question will not be tested in these sites.

## Discussion

### Research implementation sites

In order to identify the 20 research sites in which to implement this protocol, a request for proposals to participate in this multi-country study ran from October to December 2020. The request for proposals was posted on WHO/HRP’s web page and promoted through its email and social media channels. The call was open to research institutions—academic, governmental and non-governmental—around the world. Research institutions from low-income or lower-middle income countries were also able to apply for small grants of up to USD 20,000 to support the costs of protocol implementation. As part of the application, research sites were requested to make site-specific adaptations to the above protocol. The goal of this was both to determine that (1) applicants understood the research goal and methods well-enough to propose relevant site-specific implementation considerations, and (2) to ensure that applicants ultimately selected as research partners would already have a near-adapted protocol to submit for local ethics clearance.

Applications were independently reviewed and judged by WHO/HRP staff as well as external researchers who are members of HRP’s research project review panel (RP2), a mechanism by which the Department is able to obtain external review and feedback as to the technical merit of its research projects. Each application was reviewed by two individuals. Previous experience with cognitive interviewing was desirable though not required. Instead, partners needed to demonstrate:

- Strong proficiency in both English and (if applicable) the language in which interviews would be conducted.
- Experience with qualitative research, specifically in-depth interviews.
- Comfort with conducting detailed, person-to-person research about sexual *behaviours* (e.g. types of sexual activity or partners).

The call resulted in 35 applications, of which 20 were ultimately selected. This study will take place between 2021 and 2022, in collaboration with research partners in: Australia, Bangladesh, Botswana, Brazil, Canada, Colombia, Ghana, Guinea, Indonesia, Italy, Kenya, Malaysia, Mali, Mozambique, Myanmar, Nigeria, Pakistan, Thailand, Uganda, and Uruguay.

### Effect of this study

The clearest and most widely usable output of this research will be a revised survey instrument. The finalized instrument is ultimately planned for publication as a WHO/HRP resource, openly accessible for use by researchers around the world. Per the recommendations of the 2019 expert group meeting, the development of a WHO survey instrument to assess sexual health-related practices and behaviours will introduce needed comparable measures to the SRHR and HIV research communities. Increasing the availability and comparability of these data, as well as awareness around the importance of collecting it, will better enable health systems to plan for and tailor SRHR services to those who need them.

An important, second output from this research will be the site-specific results as well as the research implementation learnings that come from conducting cognitive interviews on this survey instrument among the general population in a wide variety of geographic and cultural environments. This study’s inclusion of groups that have been overlooked by SRHR research (men) or considered un/asexual (younger and older populations) challenges traditional assumptions about sexual activity and sexuality and may promote inclusion of these populations in future research.

Finally, cognitive interviewing has been an underutilized qualitative method in global health survey research [19]. The hope is that this research draws attention to the importance of cognitive interviewing as an important step in the development of new survey instruments, as well as the adaptation of existing instruments to new languages or cultural settings.

## Supplementary Information

**Additional file 1.** Draft survey instrument development—an overview.

**Additional file 2.** Sexual health survey instrument version 15, (16 November 2021).

**Additional file 3.** Cognitive interview format options: Proposed modifications for research during the COVID-19 pandemic.

## Data Availability

Not applicable.

## Abbreviations

APHRC: African Population and Health Research Center
CCCI: Cross-cultural cognitive interviewing
CI: Cognitive interview
CNEST: Cross National Error Source Typology
JAM: Joint analysis meeting
LMIC: Low- and middle-income countries
LSHTM: London School of Hygiene & Tropical Medicine
NATSAL: British National Surveys of Sexual Attitudes and Lifestyles
PI: Principal investigator
RFP: Request for proposals
RP2: WHO HRP Review Panel on Research Projects
SDG: Sustainable Development Goal
SIHI: LSHTM’s Social Innovation in Health Initiative
SRHR: Sexual and Reproductive Health and Research
WHO ERC: World Health Organization’s Ethics Review Committee
WHO/HRP: WHO’s Department of Sexual and Reproductive Health and Research, which includes the UNDP/UNFPA/UNICEF/WHO/World Bank Special Programme of Research, Development and Research Training in Human Reproduction

## Footnotes

1 This is in line with adolescent sexual and reproductive health research considerations, which indicate that “…For example, for reasons of sensitivity, such as discussions about sexual activities, substance abuse, sexual abuse, physical abuse or neglect—it may be desirable and ethically justifiable for minors (especially minors aged 16 years and older) to choose independently (without parental assistance) whether to participate in research. In this regard, minors may be unwilling to participate in the proposed research if they are required to tell their parents or guardians about the nature of the research.”[23].

## References

[1] World Health Organization (2006). Defining sexual health: report of a technical consultation on sexual health 28–31 January 2002, Geneva.

[2] Dieleman JL, Haakenstad A, Micah A, Moses M, Abbafati C, Acharya P (2018). Spending on health and HIV/AIDS: domestic health spending and development assistance in 188 countries, 1995–2015. Lancet.

[3] Starrs AM, Ezeh AC, Barker G, Basu A, Bertrand JT, Blum R (2018). Accelerate progress—sexual and reproductive health and rights for all: report of the Guttmacher–Lancet Commission. Lancet.

[4] Erens B, Phelps A, Clifton S, Mercer CH, Tanton C, Hussey D (2014). Methodology of the third British National Survey of Sexual Attitudes and Lifestyles (Natsal-3). Sex Transm Infect.

[5] Bajos N, Bozon M. Sexuality in France: practices, gender and health. Oxford: The Bardwell Press; 2012.

[6] Global Sex Survey United Kingdom: Durex; 2020 [cited 2020 Aug 3]. Available from: https://www.durex.co.uk/pages/global-sex-survey.

[7] The DHS Program. DHS Overview. Rockville: The DHS Program, USAID; 2020 [cited 2020 Aug 3]. Available from: https://dhsprogram.com/What-We-Do/Survey-Types/DHS.cfm.

[8] Postmus JL, Nikolova K, Lin HF, Johnson L (2021). Women's economic abuse experiences: results from the UN multi-country study on men and violence in Asia and the Pacific. J Interpers Violence.

[9] Human Rights Watch. Human rights watch 2017 world report: events of 2016. New York: Seven Stories Press; 2017.

[10] Lusti-Narasimhan M, Beard JR (2013). Sexual health in older women. Bull World Health Organ.

[11] Kpokiri EE, Wu D, Srinivas ML, Anderson J, Say L, Kontula O (2021). Development of an international sexual and reproductive health survey instrument: results from a pilot WHO/HRP consultative Delphi process. Sex Transm Infect.

[12] Tourangeau R, Jabine T, Straf M, Tanur J, Tourangeau R (1984). Cognitive sciences and survey methods. Cognitive aspects of survey methodology: building a bridge between disciplines.

[13] Beatty PC, Willis GB (2007). Research synthesis: the practice of cognitive interviewing. Public Opin Q.

[14] Behr D, Braun M, Dorer B. Measurement instruments in international surveys. Mannheim: GESIS—Leibniz Institute for the Social Sciences; 2016.

[15] Miller K, Fitzgerald R, Padilla J-L, Willson S, Widdop S, Caspar R (2011). Design and analysis of cognitive interviews for comparative multinational testing. Field Methods.

[16] Fitzgerald R, Widdop S, Gray M, Collins D (2011). Identifying Sources of Error in Cross-national Questionnaires: application of an error source typology to cognitive interview data. J Off Stat.

[17] Willis GB (2015). The practice of cross-cultural cognitive interviewing. Public Opin Q.

[18] Massey M. The development and testing of a module on child functioning for identifying children with disabilities on surveys. II: question development and pretesting. Disabil Health J. 2018;11(4):502–9. 10.1016/j.dhjo.2018.06.006 PMC6507431 30049637

[19] Scott K, Gharai D, Sharma M, Choudhury N, Mishra B, Chamberlain S (2020). Yes, no, maybe so: the importance of cognitive interviewing to enhance structured surveys on respectful maternity care in northern India. Health Policy Plan.

[20] Corteen E, Lapham C, Mandalia D, Clifton S, d’Ardenne J, Sadler K (2019). Question testing for the National Survey of Sexual Attitudes and Lifestyles 4: report on findings from cognitive interviews.

[21] Behr D, Braun M, Kaczmirek L, Bandilla W (2014). Item comparability in cross-national surveys: results from asking probing questions in cross-national web surveys about attitudes towards civil disobedience. Qual Quant.

[22] Fowler S, Willis GB. The practice of cognitive interviewing through web probing. In: Advances in questionnaire design, development, evaluation and testing. Hoboken: Wiley; 2020. p. 451–69.

[23] World Health Organization (2018). Guidance on ethical considerations in planning and reviewing research studies on sexual and reproductive health in adolescents.

[24] Ridolfo H, Miller K, Maitland A (2012). Measuring sexual identity using survey questionnaires: how valid are our measures?. Sex Res Soc Policy.

[25] Aicken CRH, Gray M, Clifton S, Tanton C, Field N, Sonnenberg P (2013). Improving questions on sexual partnerships: lessons learned from cognitive interviews for Britain's third National Survey of Sexual Attitudes and Lifestyles (“Natsal-3”). Arch Sex Behav.

[26] Council for International Organizations of Medical Sciences (2016). International ethical guidelines for health-related research involving humans.

[27] World Health Organization (2016). Ethical and safety recommendations for intervention research on violence against women: building on lessons from the WHO publication putting women first: ethical and safety recommendations for research on domestic violence against women.

