# Supplementary material for: Cognitive testing of a survey instrument to assess sexual practices, behaviours, and health outcomes: a multi-country study protocol
Source: Reprod Health. 2021 Dec 19;18:249. doi: 10.1186/s12978-021-01301-w (PMC8684588; doi:10.1186/s12978-021-01301-w)
Supplement: Supplementary file 1 — Additional file 1. Draft survey instrument development—an overview. [file 12978_2021_1301_MOESM1_ESM.docx]

## Additional 1 Draft survey instrument development – an overview

From September to November 2019, WHO/HRP in collaboration with a London School of Hygiene and Tropical Medicine social innovation research team organized an open call to solicit examples of existing survey instruments, domains related to sexual health, implementation considerations, and creative ideas for related measures or analyses [1]. The call was promoted on WHO/HRP’s website, through its networks, and over social media. A special session at the 24th Congress of the World Association for Sexual Health in Mexico City, Mexico also expanded the reach of the call. Submissions were accepted in all six official United Nations languages. In total, 175 submissions were received, covering all six WHO geographic regions. Of these, 139 of these were deemed eligible and graded by a team of 12 judges, resulting in 46 semi-finalists. These were reviewed by a global steering committee and the top submissions were identified.

In January 2020, using funding from WHO/HRP and the HRP Alliance, the African Population Health and Research Centre (APHRC) hosted a 3-day hackathon to fully develop the WHO/HRP standard instrument. Researchers who authored the top 18 submissions in the open call were invited as participants. A further eight experts were invited to guide discussions as facilitators. The hackathon was co-led by the LSHTM team and WHO/HRP. Over three days, a short draft instrument of priority measures was developed, with sections on: socio-demographics, sexual biography, sexual practices, sexual health-related outcomes, and social norms. Criteria for included measures are described in Box 1. [1] Key implementation considerations specific to conducting sexual and reproductive health and rights (SRHR)-related research were also identified and described in detail. Finally, opportunities were identified for: 1) development of a long(er) survey module, which could include additional relevant domains and measures; 2) development and validation of new sexual health-related indicators (for example around sex-related social norms, sexual wellbeing, etc), in the absence of existing stand-alone or short-scale measures.

In January-February hackathon participants provided final feedback on the draft instrument through a modified Delphi exercise. The refined draft was then sent for external review to approximately 70 individuals who had made eligible submissions to the open call, and 41 provided feedback. The instrument was also reviewed by relevant focal persons in WHO’s Department of Sexual and Reproductive Health and Research, to confirm that proposed measures were in line with current practice for specific SRHR domains (e.g. abortion, sexually transmitted infections,). Following refinement, the instrument was posted online in an open call for final comments, which ran from October to December 2020. Based on the revisions from this open call, the instrument was deemed ready for field testing. A manuscript, describing the above development of the short survey module and containing this latest version of the draft instrument was published. [1]

1. Kpokiri EE, Wu D, Srinivas ML, Anderson J, Say L, Kontula O, et al. Development of an international sexual and reproductive health survey instrument: results from a pilot WHO/HRP consultative Delphi process. Sexually Transmitted Infections. 2021:sextrans-2020-054822.
