# Supplementary material for: Cognitive testing of a survey instrument to assess sexual practices, behaviours, and health outcomes: a multi-country study protocol
Source: Reprod Health. 2021 Dec 19;18:249. doi: 10.1186/s12978-021-01301-w (PMC8684588; doi:10.1186/s12978-021-01301-w)
Supplement: Supplementary file 2 — Additional file 2. Sexual health survey instrument version 15, (16 November 2021). [file 12978_2021_1301_MOESM2_ESM.docx]

**Title: Sexual Health Research Survey**

**Version 15, (16 November 2021)**

**[READ TO PARTICIPANT]**

This survey is about sexual and reproductive health experiences. This information will be used to inform health policy, improve health care and health outcomes. This survey is designed to be completed by a wide range of people and so some questions may not apply to you. Some of the questions may surprise you, may cause embarrassment, and/or may be difficult to answer. Please remember you can choose not to answer any question you do not want to. All your responses will be completely confidential and kept anonymous^*^. We thank you for your participation.

**If there is a limit to confidentiality due to mandatory reporting requirements, remind participants of what must be reported.*

## A: Socio-demographics & health [INTERVIEWER ADMINISTERED]

[A1] At birth, were you described as….

1. male
2. female
3. intersex, undetermined, or another sex?

[A2] Today, do you think of yourself as…

1. man/boy
2. woman/girl
3. in another way (please specify)?

[A3] How old were you at your last birthday?

XXX years or 999 for Don’t know

[A4] Are you at present…

1. single
2. married
3. separated but still legally married
4. divorced
5. widowed?

[A5] Are you currently living with someone as a couple?

1. Yes
2. No
3. Not sure
4. Prefer not to answer

[A6] How many times have you been married or lived together with someone as a couple?

XXX times or 000 for Have never been married or lived together with someone as a couple

*[skip to A8 if answer to A6 is 000]*

[A7] How old were you when you first started living with a partner or spouse?

XXX years or 999 for Don’t know

[A8] Thinking about your health currently, how is your health in general? Is it…

1. very good
2. good
3. fair
4. poor
5. very poor?

[A9] Do you currently have any mental or physical illness or disability that affects you in your everyday life? By affecting your life, we mean limiting your usual activities in any way.

1. Yes. If yes, please list the illnesses and/or disabilities.
2. No

## B. Sexual health outcomes [INTERVIEWER ADMINISTERED]

**[READ TO PARTICIPANT]**

The next section asks about pregnancy and other sexual health outcomes.

[B1] *[to participants responding ‘female’ to A1]*

To the best of your knowledge, how many times have you been pregnant to date?

XXX or 999 for Don’t Know

[B1] *[to participants responding ‘male’ to A1]*

To the best of your knowledge, how many times have you gotten someone pregnant to date?

XXX or 999 for Don’t Know

[B2] [*only ask participants who reported 1 or more pregnancies at B1 AND responded ‘female’ to A1]:*

How old were you at the time of your **first** pregnancy (including any pregnancies that did not result in a live birth)? (age at the end of the pregnancy)

XXX years or 999 for Don’t Know

[B2] [*only ask participants who reported 1 or more pregnancies at B1 AND responded ‘male’ to A1]:*

How old were you the **first** time you got someone pregnant (including any pregnancies that did not result in a live birth)? (age at the end of the pregnancy)

XXX years or 999 for Don’t Know

[B3] [*only ask participants who reported 2 or more pregnancies at B1 AND* responded ‘female’ to A1]

How old were you at the time of your **last (or current)** pregnancy (including any pregnancies that did not result in a live birth)? (age at the end of the pregnancy)

XXX years or 999 for Don’t Know

[B3] [*only ask participants who reported 2 or more pregnancies at B1 AND* responded ‘male’ to A1]

How old were you the **last** time you got someone pregnant (including any pregnancies that did not result in a live birth)? (age at the end of the pregnancy)

XXX years or 999 for Don’t Know

[B4] [*only ask participants where A3-B3 ≤ 5 AND responded ‘female’ to A1]* When you became pregnant with your last (or current) pregnancy, how much did you personally want to become pregnant at that time? Did you…

1. not want **at all**to become pregnant at that time
2. somewhat did **not** **want** to become pregnant at that time
3. unsure about **wanting** to become pregnant at that time
4. somewhat **wanted** to become pregnant at that time
5. wanted **very much** to become pregnant at that time?

[B4] [*only ask participants where A3-B3 ≤ 5 AND responded ‘male’ to A1]* Thinking back to the last time you got someone pregnant, how much did you personally want to get them pregnant at that time? Did you…

1. not want **at all**to get them pregnant at that time
2. somewhat did **not** **want** to get them pregnant at that time
3. unsure about **wanting to get** them pregnant at that time
4. somewhat **wanted** to get them pregnant at that time
5. wanted **very much** to get them pregnant at that time?

[B5] Now I will ask you about the [insert response to B1] pregnancies you mentioned earlier:

[B5.1] [*participants who reported 1 or more pregnancies at B1 AND responded ‘female’ to A1]* First, are you currently pregnant?

1. Yes
2. No

[B5.1] [*participants who reported 1 or more pregnancies at B1 AND responded ‘male’ to A1]* First, is anyone currently pregnant with your child?

1. Yes
2. No

[B5] [*If reported* *1 or more pregnancies at B1]* How many of these pregnancies resulted in:

[B5.2] Live birth (baby born alive)

XXX or 999 for Don’t Know

[B5.3] Abortion (medical or surgical for any reason)

XXX or 999 for Don’t Know

[B5.4] Miscarriage at:

[B5.4.1] < 12 weeks pregnancy

XXX or 999 for Don’t Know

[B5.4.2] ≥12 weeks pregnancy

XXX or 999 for Don’t Know

[B5.4.3] How many required an additional medication or procedure?

XXX or 999 for Don’t Know

[B5.5] Still birth or baby born without heartbeat/not breathing

XXX or 999 for Don’t Know

[B6] [*only ask females who reported 1 or more live and/or stillbirth at B5]* How old were you when you **first** gave birth?

XXX years or 999 for Don’t know

[B6] [*only ask males who reported 1 or more live and/or stillbirth at B5]* How old were you when your first biological child was born?

XXX years or 999 for Don’t know

[B7] Have you ever had a time lasting 1 year or longer when you and your partner were trying to get pregnant and it did not happen?

1. Yes
2. No

**[READ TO PARTICIPANT]**

The following questions ask about the human immunodeficiency virus, also known as 'HIV' the virus that causes AIDS. They also ask about sexually transmitted infections, also known as STIs. As a reminder, you do not need to share any information if you do not want to.

[B8] When, if ever, were you last **tested** for HIV? Was it…

1. in the last year
2. more than 1 year ago
3. never
4. don’t know/don’t remember
5. prefer not to say?

*[Skip to B10 if answer to B8 is ‘Never’]*

[B9] What was the result of your last HIV test? Would you say…

1. I have HIV
2. I do not have HIV
3. I am still waiting for the test results
4. I don’t know/don’t remember
5. I prefer not to say?

[B10] **Aside from HIV**, when, if ever, were you last **tested** for any sexually transmitted infections (STIs) (e.g. gonorrhoea, chlamydia, syphilis, herpes, trichomoniasis, etc)? Was it…

1. in the last year
2. more than 1 year ago
3. never
4. don’t know/don’t remember
5. prefer not to say?

[B11] **Aside from HIV**, when, if ever, have you received **treatment** for any STI (e.g. gonorrhoea, chlamydia, syphilis, herpes, trichomoniasis, etc) This can be self-treatment or treatment from a doctor. Was it…

1. in the last year
2. more than 1 year ago
3. never
4. don’t know/don’t remember
5. prefer not to say?

**[READ TO PARTICIPANT]**

These next questions are about non-consensual sexual situations that you may have encountered. We understand that these are sometimes difficult to think/talk about, and you can skip any questions you feel uncomfortable answering.

[B12] Currently, in your everyday life (i.e., at work, on the street, at home), how safe do you feel from sexual assault?

1. Not at all safe

2. Somewhat unsafe

3. Neither safe or unsafe

4. Somewhat safe

5. Completely safe

777. It varies or unsure

[B13] Have you ever been either forced or frightened by another person into doing something sexually that you did not want to do?

1. Yes
2. No [*Proceed to module C*]
3. Don’t know/Cannot remember [*Proceed to module C*]
4. Prefer not to say [*Proceed to module C*]

[B14] Has this happened to you more than once?

1. Yes [*Proceed to B14.1 and B14.2*]
2. No [*Proceed to B14.3*]
3. Prefer not to say [*Proceed to module C*]

*If ‘yes’ to B14, then ask B14.1 and B14.2*

[B14.1] How old were you the **first** time this happened?

XXX years or 999 for ‘don’t know’ and 888 for ‘prefer not to say’

[B14.2] How old were you the **last** time this happened?

XXX years or 999 for ‘don’t know’ and 888 for ‘prefer not to say’

*If ‘no’ to B14, then ask B14.3*

[B14.3] How old were you when this happened?

XXX years or 999 for ‘don’t know’ and 888 for ‘prefer not to say’

## C. Sexual biography [SELF ADMINISTERED]

**[READ TO THE PARTICIPANT]**

I am now going to give you a questionnaire to complete by yourself. I won’t be able to see your answers. If you get stuck or need any help with anything let me know. Please take as much time as you need to complete the questionnaire.

**[TEXT FOR THE PARTICIPANT TO READ]**

The next question is about **sexual experience**. By ‘sexual experience' we mean any kind of contact with another person that you felt was sexual. It could be kissing, touching, intercourse, or any other form of sex.

[C1] Which of these statements best describes you? (Choose all that apply)

1. I have had sexual experiences only with males, never with females
2. I have had sexual experiences mostly with males, and at least once with a female
3. I have had sexual experiences both with males and females
4. I have had sexual experiences mostly with females, and at least once with a male
5. I have had sexual experiences only with females, never with males
6. I have (also) had sexual experience with individual(s) who are intersex, undetermined, or another sex
7. I have not had any sexual experience [*Go directly to D17*]
8. I prefer not to say

**[TEXT FOR THE PARTICIPANT TO READ]**

This next section asks questions about **sex**. By ‘sex’, we mean any sexual contact involving the genital area, including oral sex, vaginal sex, anal sex, or [*insert culturally appropriate terms].*

[C2] How old were you the first time you had sex with someone? That is, had any sexual contact involving the genital area, including oral sex, vaginal sex, anal sex, or [*insert culturally appropriate terms].* Please type in the age in years. Please estimate the age if you can’t say exactly**.**

XXX years or 999 for Don’t know. If you have never had sex type 000. [*If 000, go to D17*]

[C3] The **first** time you had sex, was the person you had sex with:

1. Male
2. Female
3. Intersex, undetermined, or another sex

[C4] The **first** time you had sex, how old was the person you had sex with? Please estimate if you do not know exactly.

XXX years or 999 for Don’t know

[C4.1] *[If C4 is 999]* Was the person you had sex with older than you, younger than you, or about the same age as you?

- 1. Older than me
  2. Younger than me
  3. About the same age as me

[C4.2] *[If C4.1 is ‘older than me’ or ‘younger than me’]* By how many years?

- 1. 1-2 years
  2. 3-5 years
  3. 6-10 years
  4. 10+ years

[C5] Which statement applies best to you the **first** time you had sex? (Choose all that apply)

1. I wanted it
2. I was forced or frightened into doing it
3. I forced or frightened the other person
4. Can’t remember

[C6] What, if any, precautions against pregnancy or HIV/STIs did either of you take the **first** time you had sex? (Choose all that apply)

1. No precautions
2. External (male) condom
3. Internal (female) condom
4. Birth control/Oral contraceptive pill
5. Morning after pill/Emergency contraceptive pill
6. IUD/Coil/Loop
7. Cap/Diaphragm
8. Injections
9. Implant
10. Spermicides (foams/gels/sprays/pessaries)
11. My partner/ I withdrew before ejaculating
12. Made sure it was safe time in my/my partner’s monthly cycle (calendar method/safe time)
13. Partner was/I had been sterilized
14. Other method of protection (please say what)
15. Don’t know

[C7] *[stratify responses by sex if reported same-sex experience at C1 or C3]* **In** **your life so far**, how many **people** have you had sex with? That is any sexual contact involving the genital area, including oral sex, vaginal sex, anal sex, or [*insert culturally appropriate terms].* Please include everyone you have ever had sex with, whether it was just once or multiple times, with a stranger, regular partner, or husband/wife.

[C8] *[stratify responses by sex if reported same-sex experience at C1 AND if reported 1 or more partners of each sex in C7]* **In the last year**, how many **people** have you had sex with? That is any sexual contact involving the genital area, including oral sex, vaginal sex, anal sex, or [*insert culturally appropriate terms]*?

[C9] *[stratify responses by sex if reported same-sex experience at C1 AND if reported 1 or more partners of each sex in C8]* **In the last 4 weeks,** how many **people** have you had sex with? That is any sexual contact involving the genital area, including oral sex, vaginal sex, anal sex, or [*insert culturally appropriate terms]*?

**[Text for participant to read]**

The next section is about situations when sex is exchanged for goods, services, or money.

[C10] When, if ever, was the last time you **gave** money, material goods, favours, gifts, drugs, or shelter in exchange for sex? By material goods, we mean things like food, rent, clothes/shoes/cell phones, cosmetics, transport, good marks in school or school fees, or items for someone or for their children, or their family.

1. In the last year
2. More than a year ago
3. Never

[C11] When, if ever, was the last time you **received** money, material goods, favours, gifts, drugs, or shelter in exchange for sex? By material goods, we mean things like food, rent, clothes/shoes/cell phones, cosmetics, transport, good marks in school or school fees, or items for your children, your family, or yourself.

1. In the last year
2. More than a year ago
3. Never

## D. Sexual Practices [SELF ADMINISTERED]

**[Text for participant to read]**

This section asks questions about sexual practices. As a reminder, the interviewer will not be able to see your answers. If you get stuck or need any help with anything let them know. Please take as much time as you need to complete the questionnaire.

[D1] *[Only ask if C8 ≥1]* In the last four weeks, how many **times** have you had sex with another person or people? That is any sexual contact involving the genital area, so including oral sex, vaginal sex, anal sex, or [*insert culturally appropriate terms].*

XXX times or 999 for Don’t know.

[D2] *[Only ask if D1 is 0]* When did you last have sex?

MM/YY

[*Only ask D3-D9 if C8 ≥1*]

**[Text for participant to read]**

Questions D3-D9 are about the **last** time you had sex, meaning the most recent time you had **any** sexual contact with another person.

[D3] Which one of these descriptions applies best to you and ***(****that person****)***at the time you **most recently**had sex? Only give one answer

1. We were living together as a couple / married at the time
2. We were in a steady relationship at the time
3. We used to be in a steady relationship, but were not at that time
4. We had known each other for a while, but were not in a steady relationship
5. We had recently met
6. We had just met for the first time

[D4] The **most recent time** you had sex, how old was the person you had sex with? Please estimate if you do not know exactly.

XXX years or 999 for Don’t know

[D4.1] *[If D4 is 999]* Was the person you had sex with older than you, younger than you, or about the same age as you?

- 1. Older than me
  2. Younger than me
  3. About the same age as me

[D4.2] *[If D4.1 is ‘older than me’ or ‘younger than me’]* By how many years?

- - 1. 1-2 years
    2. 3-5 years
    3. 6-10 years
    4. 10+ years

[D5] [*If reported same-sex experience at C1]* The most recent time you had sex, was the person you had sex with:

1. Male
2. Female
3. Intersex, undetermined, or another sex

[D6] The **most recent time** you had sex, what did you consider the ethnicity of the person you had sex with to be? < response options locally determined >

[D7] The **most recent** time you had sex with this person, which of the following did you do? (Choose all that apply)

1. You performed oral sex on them. That is, your mouth on their genital area.
2. They performed oral sex on you. That is, their mouth on your genital area.
3. You had penile-vaginal intercourse. Vaginal sex is a penis in a vagina.
4. You had **receptive** penile-anal intercourse. Receptive anal sex is having a penis inserted into **your** anus (rectum or back passage).
5. You had **insertive** penile-anal intercourse. Insertive anal sex is inserting **your** penis into another person’s anus (rectum or back passage).
6. You stimulated **their** anus or inserted something in their anus that is NOT a penis, but includes fingers, mouth, hands, dildos, toys, or other sexual aids)
7. They stimulated **your** anus or inserted something in your anus that is NOT a penis, but includes fingers, mouth, hands, dildos, toys, or other sexual aids)
8. You had manual sex. This is using fingers, hands, dildos, toys, or other sexual aids on or in a genital area.
9. Other sexual contact not listed here

[D8] What, if any, precautions against pregnancy or HIV/STIs did either of you take, the **most recent time** you had sex? (Choose all that apply)

1. No precautions
2. External (male) condom
3. Internal (female) condom
4. Birth control/Oral contraceptive pill
5. Morning after pill/Emergency oral contraceptive pill
6. IUD/Coil/Loop
7. Cap/Diaphragm
8. Injections
9. Implant
10. Spermicides (foams/gels/sprays/pessaries)
11. My partner/I withdrew before ejaculating
12. Made sure it was safe time in my/my partner’s monthly cycle (calendar method/safe time)
13. Partner has been /I have been sterilized
14. Other method of protection (please say what)
15. Don’t know

[D9] How pleasurable did **you** find the last time you had sex?

1. very pleasurable
2. pleasurable
3. neutral
4. unpleasurable
5. very unpleasurable.

[*For types of sex that did not happen when participant* ***last*** *had sex (per answer to D7), D10-16 will be asked*]

[D10] When, if ever, was the last time you performed oral sex on someone. That is, your mouth on their genital area?

1. In the last 4 weeks
2. more than 4 weeks ago, but within the last year
3. more than 1 year ago
4. never

[D11] When, if ever, was the last time someone performed oral sex on you? That is, their mouth on your genital area?

1. In the last 4 weeks
2. more than 4 weeks ago, but within the last year
3. more than 1 year ago
4. never

[D12] When, if ever, was the last time you had vaginal sex with someone? Vaginal sex is a penis in a vagina.

1. In the last 4 weeks
2. more than 4 weeks ago, but within the last year
3. more than 1 year ago
4. never

[D13] When, if ever, was the last time you had **receptive** anal sex with someone? Receptive anal sex is having a penis inserted into your anus (rectum or back passage).

1. In the last 4 weeks
2. more than 4 weeks ago, but within the last year
3. more than 1 year ago
4. never

[D14] *[to participants responding ‘male’ to A1]* When, if ever, was the last time you had **insertive** anal sex with someone? Insertive anal sex is inserting your penis into another person’s anus (rectum or back passage).

1. In the last 4 weeks
2. more than 4 weeks ago, but within the last year
3. more than 1 year ago
4. never

[D15] When, if ever, was the last time you were anally stimulated/you anally stimulated someone? Anal stimulation is stimulating or inserting something other than a penis (including hands, mouth, dildo or other sexual aids) into the anus (rectum or back passage)).

1. In the last 4 weeks
2. more than 4 weeks ago, but within the last year
3. more than 1 year ago
4. never

[D16] When, if ever, was the last time you had manual sex with someone. This is using fingers, hands, dildos, toys, or other sexual aids on or in a genital area.

1. In the last 4 weeks
2. more than 4 weeks ago, but within the last year
3. more than 1 year ago
4. never

[D17] Solo masturbation: When, if ever, did you **last** masturbate, that is, arouse and pleasure **yourself** sexually?

1. In the last 4 weeks
2. more than 4 weeks ago, but within the last year
3. more than 1 year ago
4. never

[D18] In general, how satisfied have you been with your sex life in the last year?

1. very satisfied
2. satisfied
3. neutral
4. dissatisfied
5. very dissatisfied

**[TEXT FOR THE PARTICIPANT TO READ]**

Thank you for your responses! Please return the device to the interviewer.

## E. Social Perceptions/Beliefs [INTERVIEWER ADMINISTERED]

**[READ TO PARTICIPANT]**

In this section, I will ask you questions about your beliefs about sex and sexual and reproductive health.

For E1 – E13, please read the following statements and say whether **you**:

1. Strongly agree
2. Agree
3. Disagree
4. Strongly disagree
5. Prefer not to answer

[E1] Sex education promotes sexual activity among young people.

[E2] A woman has the right to say ‘no’ to sex if she does not want it.

[E3] A man has the right to say ‘no’ to sex if he does not want it.

[E4] It is acceptable for a woman to have sex before marriage.

[E5] It is acceptable for a man to have sex before marriage.

[E6] Having sex that is pleasurable is important for a woman’s sex life and general well being

[E7] Having sex that is pleasurable is important for a man’s sex life and general well being

[E8] Sex between two consenting adult women is always wrong.

[E9] Sex between two consenting adult men is always wrong.

[E10] Men naturally have more sexual needs than women.

[E11] It is okay for a woman to use a modern contraceptive method/family planning (*e.g.* birth control/oral contraceptive pills, injection, implants, loop or coil (IUD), condoms, etc) to avoid or delay pregnancy if she wishes.

[E12] It is okay for a woman to [have an abortion / terminate a pregnancy] if she does not want to have a child

[E13] Who do you think should decide whether a woman [has an abortion/ terminates a pregnancy]? Is it…

1. Mainly her decision
2. Mainly her husband’s or partner’s decision
3. They should decide together
4. Others (please specify)
5. Nobody
6. Prefer not to answer?

## F. Identity and Rights [SELF ADMINISTERED]

**[READ TO THE PARTICIPANT]**

I am now going to give you the questionnaire to complete by yourself. As before, I won’t be able to see your answers. If you get stuck or need any help with anything let me know. Please take as much time as you need to complete the questionnaire.

**[TEXT FOR THE PARTICIPANT TO READ]**

This section will ask you questions about **your rights and identity**.

[F1] Do you think of yourself as ...

1. Heterosexual or straight

2. Gay, lesbian, or homosexual

3. Bisexual

4. Pansexual

5. Asexual

6. Not sure; undecided /another identity not listed here

7. Prefer not to answer

[F2] Have you ever been discriminated against because of your **sexual orientation**? *Sexual orientation refers to a person’s physical, romantic, and/or emotional attraction towards other people.*

1. Yes
2. No [*Go to F3*]

[F2.1] When was the last time you were discriminated against?

1. In the last year
2. More than 1 year ago
3. Don’t know
4. Prefer not to answer

[F3] For the following 9 statements (A-I), please mark when, if ever, you have experienced any of the following on the grounds of your **sexual orientation**?

1. In the last year

2. More than 1 year ago

3. Never

4. Don’t know/prefer not to answer

[F3.1] I have been insulted or threatened.

[F3.2] I have been beaten, pushed or kicked

[F3.3] My belongings have been destroyed or damaged

[F3.4] I was not given a job or was dismissed from my job

[F3.5] I was treated in a discriminatory way by a healthcare professional

[F3.6] I was denied medical treatment

[F3.7] I was jailed, prosecuted or denied legal services

[F3.8] I was asked to leave my home or thrown out of my accommodations

[F3.9] I was forced to engage in a sexual act, sexually assaulted, or raped

[F4] Have you ever been discriminated against because of your **gender identity**? *Gender identity refers to someone’s individual and internal experience of gender, which may or may not correspond with their sex assigned at birth.*

1. Yes
2. No [*Go to F5*]

[F4.1] When was the last time you were discriminated against?

1. In the last year
2. More than 1 year ago
3. Don’t know
4. Prefer not to answer

[F5] For the following 9 statements (A-I), please mark when, if ever, you have experienced any of the following on the grounds of your **gender identity**?

1. In the last year

2. More than 1 year ago

3. Never

4. Don’t know/prefer not to answer

[F5.1] I have been insulted or threatened.

[F5.2] I have been beaten, pushed or kicked

[F5.3] My belongings have been destroyed or damaged

[F5.4] I was not given a job or was dismissed from my job

[F5.5] I was treated in a discriminatory way by a healthcare professional

[F5.6] I was denied medical treatment

[F5.7] I was jailed, prosecuted or denied legal services

[F5.8] I was asked to leave my home or thrown out of my accommodations

[F5.9] I was forced to engage in a sexual act, sexually assaulted, or raped

[F6] Education < response options locally determined >

[F7] Which best describes your employment?  < response options locally determined >

[F8] How often does your household not have enough resources to obtain what it needs to live day to day?

1. Every day
2. At least once per week
3. At least once per month
4. At least once per year
5. Never

[F9] To which of the following ethnic groups do you consider you belong? < response options locally determined >

[F10] What is your current religion? < response options locally determined >

[F10.1] How important are religion and religious beliefs to you now?

1. Not important at all
2. Not very important
3. Fairly important
4. Very important

Thank you for completing the survey. [THE END]

**[NOTE FOR IMPLEMENTATION]**

A site-specific end script thanking the participant for coming, letting them ask questions, and offering additional specific information about services or (where necessary) providing referrals should be put here.
