# Supplementary material for: Cognitive testing of a survey instrument to assess sexual practices, behaviours, and health outcomes: a multi-country study protocol
Source: Reprod Health. 2021 Dec 19;18:249. doi: 10.1186/s12978-021-01301-w (PMC8684588; doi:10.1186/s12978-021-01301-w)
Supplement: Supplementary file 3 — Additional file 3. Cognitive interview format options: Proposed modifications for research during the COVID-19 pandemic. [file 12978_2021_1301_MOESM3_ESM.docx]

# Additional File 3 - Cognitive interview options

## Proposed modifications for research during COVID-19

The proposed WHO Sexual Health Survey questionnaire will be cognitively tested in low-, middle-, and high-income countries during 2021. The aims of the cognitive testing are: to establish whether the target audiences (cross sections of the general population) are willing and able to answer the proposed questions; and whether questions are interpreted (understood) by the target population in the way intended.

The ongoing COVID-19 pandemic means that the protocol for cognitive testing needs to allow for the possibility that cognitive testing may not be possible face-to-face. Three options are considered:

1. Covid-safe Face-to-face – where interviews take place in accordance with local social distancing rules
2. Remote cognitive interviewing through video-conferencing software such as Teams, Zoom, etc.
3. Web probing (i.e. no interviewer involvement or interviewer briefs participant on what to do, encourages participation by phone/video-conference)

## Description of each option

| Covid-safe face-to-face (F2F) – where interviews take place in accordance with local social distancing rules | Cognitive interviewer introduces the testing and explains how the interview will work.  The survey is administered as it would be in practice, with the interviewer asking certain modules, while other modules are self-complete. For interviewer-administered sections, the interviewer asks probes where indicated.  For any sections designated ‘self-complete’, the interviewer will ask the participant to say when they have completed certain questions so that interviewer can ask them to answer probes asked on paper. Paper responses are put in and envelope and sealed by participant, who returns them to the interviewer. This is done to protect respondent confidentiality and to explore understanding and acceptability of the most sensitive questions.  This approach was used in the British National Survey of Sexual Attitudes and Lifestyles (Natsal 4)[1] |
| --- | --- |
| Remote cognitive interviewing through video-conferencing software such as Teams, Zoom, etc. | As above, but any questions designated ‘self-complete’ are included in an electronic version of the questionnaire (EQ) that is shared with the participants at the start of the interview.  Web probes (sometimes referred to as random probing) are included in the EQ, either immediately following a survey question or at the end of the module. |
| Web probing (no interviewer involvement or interviewer briefs participant on what to do, encourages participation by phone/video-conference) | All probes are designed as web probes and embedded within the test questionnaire. All test questions are administered as self-completion. Participants complete the test questionnaire remotely, either online or offline. |

**Pros and cons of each option**

| Option | Pros | Cons |
| --- | --- | --- |
| Covid-safe F2F cognitive interviewing | Build rapport with participant that supports and encourages dialogue about the questions  Deeper exploration of issues, as interviewer can follow up with expansive or elaborative probes [2]  Affords collection of full paralinguistic response to survey questions  Can provide reassurance to participant, keep participant motivated  Reflexive: probing is tailored to the individual participant | Characteristics and behaviour of interviewer may influence how participant answers survey questions and probes  May not be feasible to undertake face-to-face interviewing due to national/local lockdowns  Expensive – interview travel costs |
| Remote cognitive interviewing via video-conference | Benefits of F2F interviewing but without need for F2F contact, thus eliminating risk of coronavirus transmission  Affords option to share screen, so that both interviewer and participant can view survey questions/answer option etc.  Reduced fieldwork costs as no travel | Risk of technology failure  Excludes those without internet connectivity, as well as those who are unfamiliar or uncomfortable with using video-conferencing technology |
| Web probing | Can undertake cognitive testing remotely, with minimal risk of infection and with no field costs [3]  Open-ended, embedded probes can uncover the same types of issues as traditional cognitive interviewing [4, 5]  Standardised probes across countries/cultures supports comparability [6]  Participants can work through the survey questions and follow up questions at their own pace  Participants may be more honest and open in their responses to probes, when completing online | Probes need to be programmed into the test questionnaire. This will be an additional cost and add to the test instrument development time.  Probes can suffer from the same problems as open survey questions [6]  Loss of reflexivity - probes cannot be tailored to the individual participant in the way they can by an interviewer [7] and information may be lost  Inclusion adds to response burden, and as such the number of probes is limited. For example, Schuman (1966) targeted only 10 survey questions with 10 follow up probes.[8] Cannell *et al.* (1989) evaluated 20 survey items in a split ballot design. [9]  Restricts testing to those who have access to the internet or are comfortable using an electronic device [6] |

# Conclusions

The questions being tested are potentially sensitive and standard cognitive interviewing protocols, based on verbal reports, may not be appropriate for exploring understanding and answer processes for some questions (i.e. the most sensitive items contained the self-completion modules) in some contexts.

To overcome this problem Natsal-4 used scripted probes that were administered in a self-completion form on a card that was completed by the participant and returned to the interviewer in a sealed envelope. These probes took the form of closed and open questions. These follow up questions were typically asked retrospectively, once participants had completed the module.

Web probing is based on a similar idea: participants are asked scripted follow up questions that typically explore comprehension, answer-category selection and item-specific information. Guidance on implementation of web probing suggests:

Web probing generates open-text responses that are typically full sentences and participants do respond to expansive probes (designed to generate additional explanation) in contexts where this approach has been used.

Web probing appears to generate comparable findings with traditional cognitive interviewing.

Probes should be worded in a way that clearly communicates to participants what is expected of them.

There is mixed advice on the placement of probes – embedded (concurrent) or retrospective. Fowler and Willis found ‘evidence that probing immediately after the item produced more salient responses to the probes than the retrospective condition’. [5] They also found no adverse impact on substantive answers due to the immediate placement of probes. Behr and colleagues advocate the use of embedded probes.

Behr and her colleagues have also used web probing to look at construct or item bias in cross-national studies. [6, 10, 11]. Authors note that in cross-national and cross-cultural cognitive interviewing research not all probe types work equally well across all groups [12] and that the use of web probing beyond ‘Western’ country contexts or with populations in which ‘survey and opinion research is not widely known or used’ needs careful consideration.[6]

It is also worth noting that web probing is often advocated as part of a mixed methods approach to questionnaire development and testing, with web probes being devised on the basis of findings from earlier rounds of cognitive interviewing. In the context of testing the WHO Sexual Health questionnaire, such an approach is unlikely to be feasible. A pragmatic approach is needed in which the impact of the current COVID-19 pandemic on both research budgets and interviewer and participant safety are balanced with the need to pretest the new questionnaire.

Various authors note that analysis of web probing findings is time-consuming, though it is not clear that it is any more time-consuming than undertaking rigorous analysis of cognitive interview data. Thought is needed about who will undertake the analysis of web probes. In the literature this has tended to be a central research team. However, for the WHO Sexual Health survey instrument testing, the local teams could undertake this analysis, using a similar protocol to that used for analysis of the cognitive interviews.

Some of the questions to be tested are designed to be administered by an interviewer. To use the web probing approach would involve them being administered in a self-completion format. This change of mode could introduce a risk of measurement error. This should be born in mind when interpreting findings and making comparisons with findings from face-to-face cognitive interviews. To assess the risk of measurement error, analysts could use the tool developed by d’Ardenne and colleagues. [13]

A suggested approach is:

- Use similar probes in both the cognitive interviews and web probing to aid comparability of findings.
- Some cognitive interview probes can be read out verbatim to aid comparability of findings across countries. This will support inexperienced cognitive interviewers. However, within the cognitive interview there should be flexibility for interviewers to use expansive and elaborative probes to explore responses further. [12]
- Provide guidance to translators. Guidance should take the form of definitions and or clarification of key concepts being measured by the question(s), especially where the words themselves are unlikely to have direct equivalence in other languages.
- If possible test the interview protocols with a few participants in at least two countries and make any refinements to the protocol before rolling out further. [14, 15]
- Remote cognitive interviews – the feasibility and cultural acceptability of this option in individual countries should be explored. Given that the visual presentation of answer options (in interview-administered modules) and questions and response options (in self-completion modules), the use of video conferencing involving both video and voice is important. It allows the sharing of visual material with the participant and for visual confirmation that the participant has viewed the material. This would not be possible through voice alone (or by telephone). If video is not feasible then the protocol should advise that the interview not take place.
- Test the source language questions first, to provide ‘a measure of baseline questionnaire functioning by which to assess the operation of the translation’.[12] This is necessary for determining whether problems are related to the source questionnaire or are to do with translation or cultural portability, for example.
- Providing participants with an off-line version of the questionnaire (e.g. on an easy-to-use electronic device, or a paper version of the questionnaire with embedded probes could be considered, to improve population coverage. However, it might be more cost-effective to undertake face-to-face cognitive testing, following appropriate social distancing/ COVID-safety protocols.

**References**

1. Corteen E, Lapham C, Mandalia D, Clifton S, d’Ardenne J, Sadler K. Question testing for the National Survey of Sexual Attitudes and Lifestyles 4: Report on findings from cognitive interviews. NatCen Social Research; 2019.

2. Beatty PC, Willis GB. Research Synthesis: The Practice of Cognitive Interviewing. Public Opinion Quarterly. 2007;71(2):287-311.

3. Fowler S, Willis G, Moser R, Ferrer R, Berrigan D, editors. Use of Amazon MTurk online marketplace for questionnaire testing and experimental analysis of survey features. Federal Committee on Statistical Methodology Research Conference, Washington, DC, December; 2015.

4. Braun M. Using Egalitarian Items to Measure Men’s and Women’s Family Roles. Sex Roles. 2008;59(9):644-56.

5. Fowler S, B. Willis G. The practice of cognitive interviewing through web probing. Advances in Questionnaire Design, Development, Evaluation and Testing. 2020:451-69.

6. Behr D, Meitinger K, Braun M, Kaczmirek L. Cross-National Web Probing: An Overview of Its Methodology and Its Use in Cross-National Studies. Advances in Questionnaire Design, Development, Evaluation and Testing2020. p. 521-43.

7. Behr D, Bandilla W, Kaczmirek L, Braun M. Cognitive Probes in Web Surveys: On the Effect of Different Text Box Size and Probing Exposure on Response Quality. Social Science Computer Review. 2013;32(4):524-33.

8. Schuman H. The random probe: A technique for evaluating the validity of closed questions. American sociological review. 1966:218-22.

9. Cannell C, Fowler F., Kalton, G., et al. New quantitative techniques for prestesting surveys. 47th International Statistical Institute; Paris, France1989.

10. Meitinger K, Behr D. Comparing Cognitive Interviewing and Online Probing: Do They Find Similar Results? Field Methods. 2016;28(4):363-80.

11. Meitinger K, Braun M, Behr D, editors. Sequence matters in web probing: the impact of the order of probes on response quality, motivation of respondents, and answer content. Survey Research Methods; 2018.

12. Willis GB. The Practice of Cross-Cultural Cognitive Interviewing. Public Opinion Quarterly. 2015;79(S1):359-95.

13. d'Ardenne J.; Collins D.; Gray MJ, C.; Pilley, S. Assessing the risk of mode effects: Review of proposed survey questions for waves 7-10 of Understanding Society. NatCen Social Research 2017. Contract No.: 4.

14. Levin K, Willis GB, Forsyth BH, Norberg A, Kudela MS, Stark D, et al., editors. Using cognitive interviews to evaluate the Spanish-language translation of dietary questionnaire. Survey Research Methods; 2009.

15. Miller K, Fitzgerald R, Padilla J-L, Willson S, Widdop S, Caspar R, et al. Design and analysis of cognitive interviews for comparative multinational testing. Field Methods. 2011;23(4):379-96.
